# Two-Way Communication Digital Power Controllers for Wireless Retinal Prosthesis Systems

**DOI:** 10.3390/s22082970

**Published:** 2022-04-13

**Authors:** Ruhaifi Bin Abdullah Zawawi, Jungsuk Kim

**Affiliations:** 1 Collaborative Microelectronic Design Excellence Center (CEDEC), Universiti Sains Malaysia, SAINS@USM, Level 1 Block C, No 10, Persiaran Bukit Jambul, Bayan Lepas 11900, Malaysia; ruhaifi@usm.my; 2 R&D Laboratory, Cellico Company, 815 Daewangpangyo-ro, Seongnam-si 13449, Korea; 3 Department of Biomedical Engineering, Gachon University, 191 Hambakmoe-ro, Incheon 21999, Korea

**Keywords:** wireless power telemetry, digital power controller, retinal prosthesis, implantable medical devices

## Abstract

Power-efficient digital controllers are proposed for wireless retinal prosthetic systems. Power management plays an important role in reducing the power consumption and avoiding malfunctions in implantable medical devices. In the case of implantable devices with only one-way communication, the received power level is uncertain because there is no feedback on the power status. Accordingly, system breakdown due to inefficient power management should be avoided to prevent harm to patients. In this study, digital power controllers were developed for achieving two-way communication. Three controllers—a forward and back telemetry control unit, a power control unit, and a preamble control unit—operated simultaneously to control the class-E amplifier input power, provided command data to stimulators, monitored the power levels of the implanted devices, and generated back telemetry data. For performance verification, we implemented a digital power control system using a field-programmable gate array and then demonstrated it by employing a wireless telemetry system.

## 1. Introduction

Over the past few decades, functional electrical stimulation has restored function in neurologically impaired individuals [1]. Blindness due to retinitis pigmentosa or age-related macular degeneration causes degeneration of rods and cones in the retina. In 1755, Charles Le Roy introduced a stimulation technique based on electrically induced visual perception. It has been reported that blind patients can perceive phosphene when an electric current is applied across the ocular surface [2]. In 1980, advances in materials and microfabrication allowed scientists and engineers to conduct research on retinal prosthetics. In the early stages of implantable medical devices (IMDs), a wire passing through the skin was employed to directly provide power to the implants without power transfer loss. However, the transcutaneous connection can lead to infections in the skin and soft tissue, making the wire impractical [3]. To overcome this shortcoming, alternative power transfer methods have been introduced, such as ultrasound, near-field capacitive, inductive, and mid-field resonant couplings. As reported in [4], for a wireless system with a power budget of >100 mW, the near-field inductive coupling method is the best candidate for high-power and long-range power transmission.

For wireless IMDs, the power transfer efficiency (PTE) is of prime concern because power loss during wireless transmission can lead to electromagnetic radiation exposure and secondary heating effects [5]. The PTE is largely determined by the coupling coefficient of the inductive link but is also affected by the battery, power amplifier switching circuit, tissue status, and power conditioning, e.g., the alternating current (AC)–direct current (DC) and DC–AC converters [6]. However, the secondary coil geometry of the inductive-coupled coils is limited by the shape of the eyeball, which has a diameter of approximately 2.5 cm. This small coil dimension reduces the quality factor of the coil, which is proportional to the coupling coefficient; thereby reducing the total PTE. To compensate for the poor PTE, various coil optimization techniques for near-field inductive-coupling power transmission have been proposed [5,7,8,9,10,11]. However, the PTE is still reduced by coil misalignment inside the eyeball, which often occurs after implantation or during eye rolling [12,13]. This causes a power disruption; thus, the implanted system can break down because of an insufficient power supply. Therefore, power control units (PCUs) are indispensable in IMDs—particularly retinal implants that consume high power—to monitor prosthetic device operation and efficiently manage the power distribution. For example, the PCU can observe the rectified voltage in the IMD to determine whether it exceeds the safe voltage level of the receiver. When the IMD receiver enters a worse power condition, the PCU turns off several functional blocks, saving power and preventing malfunctions of the IMD. Subsequently, the PCU in the receiver (Rx) provides the error data to the transmitter (Tx). Consequently, the Tx can adjust the wireless power transmission supply in real time. The two-way communication between the Tx and Rx allows sufficient power delivery to the implanted devices.

Motivated by this, we propose a methodology for maintaining the power of implanted devices at a safe level. To realize this function for retinal prostheses, three main controllers were proposed, designed, and demonstrated. The first controller implemented in the transmitter, i.e., the forward and back telemetry control unit (FBCU), processes the error data received from the implanted device and manages the input power of the class-E amplifier. During normal operation, the command data are generated by this controller. The second and third controllers, i.e., the PCU and preamble control unit (PRCU), deliver the command data used to decide the stimuli current waveforms to the stimulators, track the power level, and send error data back to the transmitter when the power supply is insufficient.

This paper extends our previous work in [14,15]. The system platform proposed in the previous work is a one-way communication system. In this system, the power and data are wirelessly transferred via an inductive link. When the system operates, the rectified voltage in the receiver is observed through an oscilloscope so that the regulator receives sufficient voltage from the rectifier. When the rectified voltage drops because of the variation in output load, the power level in the transmitter is increased by manually adjusting the supply voltage. The problem will occur when the receiver is implanted. In this condition, the power level in the receiver becomes unknown. With the proposed digital power controllers, the mentioned issues are resolved, whereby the power level in the receiver is continuously observed and the transmitter responds to the power level condition of the receiver. This ensures that the operation of the system platform is more reliable. 

The remainder of this paper is organized as follows. Section 2 presents an overview of the wireless retinal prosthetic system, along with a system flow diagram. The system implementation is discussed in this section. Section 3 presents the simulation and measurement results. Finally, the conclusions are presented in Section 4.

## 2. Method

### 2.1. Overview of the Wireless Power Telemetry System

**Figure 1:**
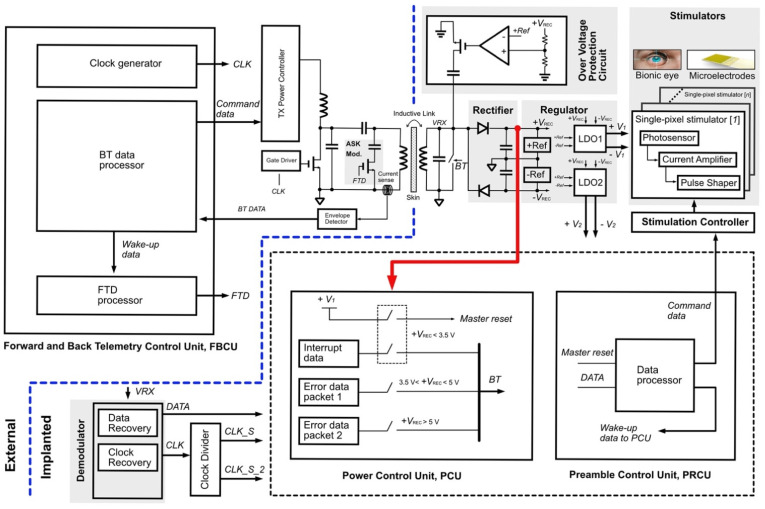
Wireless power retinal prosthesis system.

Figure 1 shows the architecture of the wireless power telemetry system. The external devices consist of a class-E power amplifier, an amplitude-shift keying (ASK) modulator, a current-sensing circuit, the FBCU, and a Tx power controller. The implanted side comprises a rectifier, regulators, a demodulator, an overvoltage protection circuit, the PCU, the PRCU, and stimulators.

A class-E power amplifier wirelessly transmits power to the implanted devices when both the primary and secondary coils are magnetically coupled. Consequently, the magnetic flux generated by the primary coil induces an AC voltage in the receiver coil, resulting in unregulated voltages at the rectifier circuit output. Low-dropout regulators (LDOs) generate constant dual-polarity DC voltages. The red arrow in Figure 1 indicates a connection from the positive *V*_REC_ node to the PCU. The PCU senses the level of the rectifier output voltage *V*_REC_ during the power-up and continuously sends back error data packets to the transmitter with unique bitstreams depending on the rectifier’s voltage level. The FBCU in the transmitter processes the error data; accordingly, the transmitter’s power level is adjusted, the data packet composes the header, and the command data are transmitted to the receiver, noted as *FTD*. The PRCU processes the data packet and generates input data for the stimulation controller, as well as a wake-up signal for the PCU. This process continues until the rectified voltage falls below its minimum voltage level; in this state, the transmitter enters the waiting mode while the implanted PCU senses the rectified voltage and sends an error data packet to the transmitter. Regular operation is resumed after the rectified voltage reaches the safe voltage level. The FBCU, PCU, and PRCU are described in Section 2.3.

### 2.2. Flow Diagram

**Figure 2:**
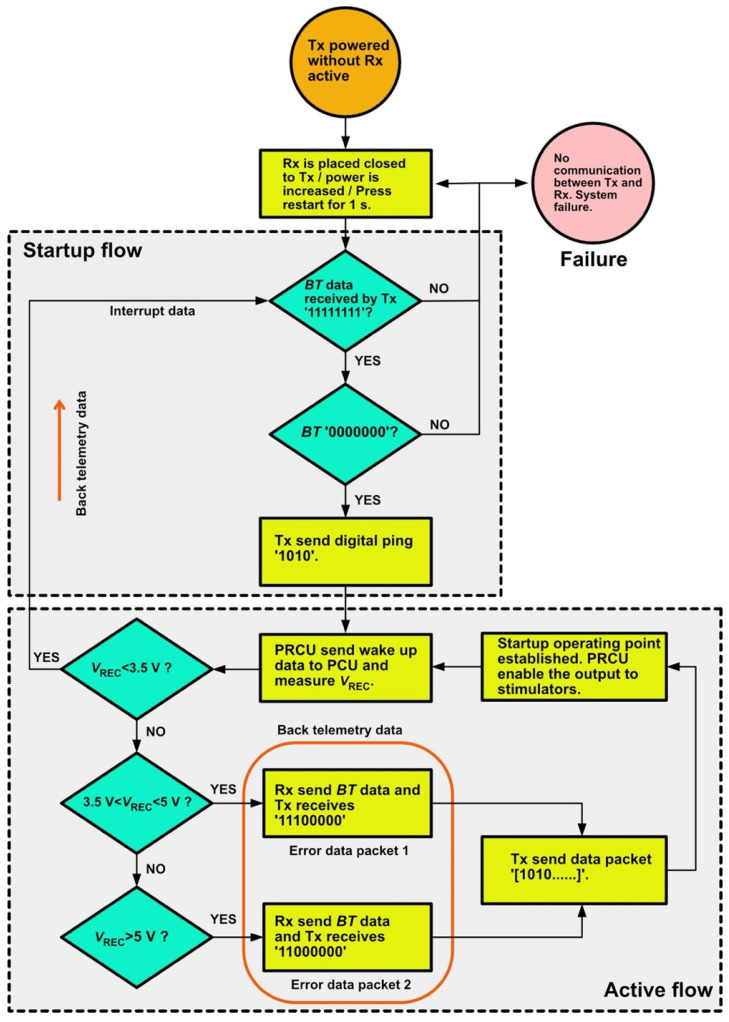
Power telemetry start-up and active flow diagram.

The detailed operation of the proposed system is shown in the flow diagram of Figure 2. This diagram illustrates the flow of the system operation from start-up to active function. During the start-up operation, the receiver coil must be placed close to the transmitter coil. Therefore, a bitstream of long “1” data is expected in the initial state, followed by long “0” data when the receiver is present. The transmitter initiates the handshake by transmitting a digital ping. The PRCU processes the ping data and generates wake-up data for the PCU.

There are three conditions of the system: (1) worst-case, (2) typical, and (3) best-case. The worst-case condition occurs when the rectified voltage *V*_REC_ is <3.5 V. The typical condition occurs when *V*_REC_ is between 3.5 and 5 V, and the best-case condition corresponds to *V*_REC_ > 5 V. The closed loop of the active operation is considered in the typical and best-case conditions only. The PCU senses the rectified voltage level during active operation and generates erroneous data packets. The data are modulated in the Rx signal using a load-shift keying modulation technique, which varies the reflected load observed by the Tx. Consequently, the current in the Tx coil changes, and the current-sensing circuit in the Tx demodulates the error data packets according to the current changes. A command data packet is then transmitted to the receiver for further processing in the stimulation controller.

All the functional blocks in the implanted system are in the sleep mode, except for the PCU, which generates interrupt data and sends them to the transmitter for the worst-case scenario. Finally, the system returns to its initial state, wherein it is necessary to adjust the T_X_ coil position or increase the power level of the class-E amplifier. System malfunction occurs when there is no communication between the transmitter and receiver.

Numerous factors may lead to system failure. In the transmitter, an unoptimized class-E amplifier that operates under a nonzero voltage switching condition may fail to deliver sufficient power to the receiver due to significant power loss in the switching transistor occurring; consequently, the rectified voltage in the receiver is low (below 3 V) and cannot drive the regulator output of 2.5 V. Under this condition, the receiver remains in the off state and fails to generate any signals. The selection of the components of the transmitter and receiver is crucial; the voltage across each component during operation should be below the maximum voltage rating to avoid device failure. In our case, the maximum voltage rating of each component is 50 V. In addition, it is critical for wireless transmission to operate at the resonance frequency, 13.56 MHz, to maximize the PTE so that the transmitter can deliver sufficient power to the receiver.

### 2.3. System Implementation

**Figure 3:**
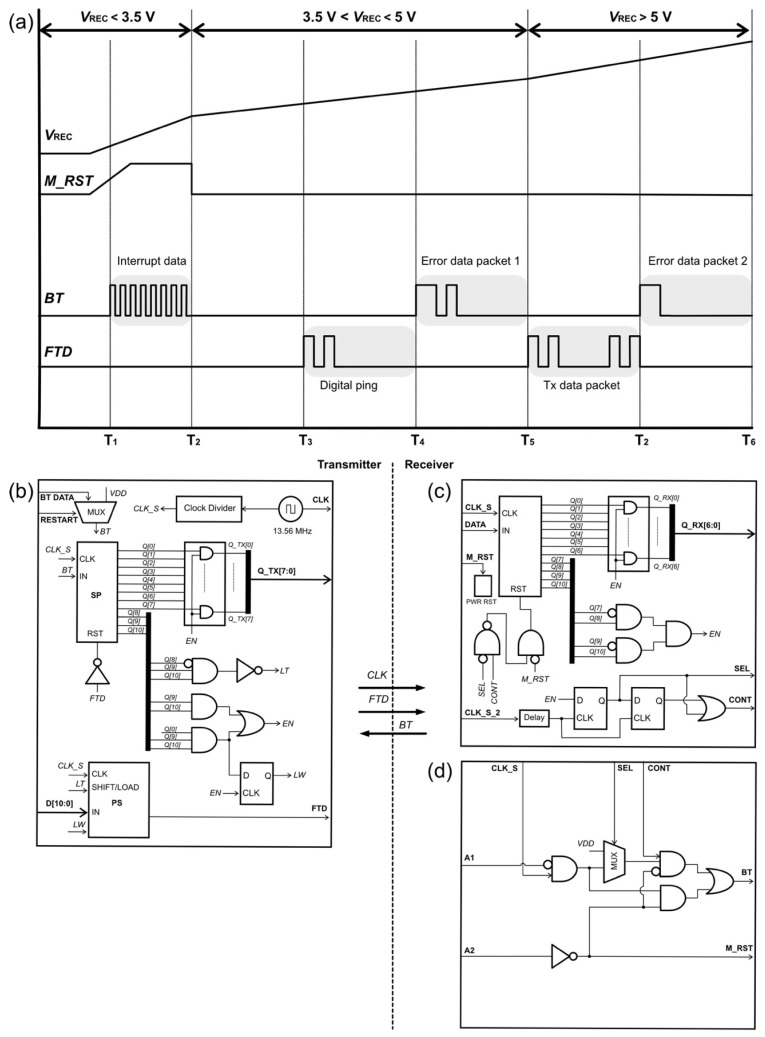
(**a**) Timing diagram of the digital controllers; (**b**) FBCU, (**c**) PRCU, and (**d**) PCU.

**Figure 4:**
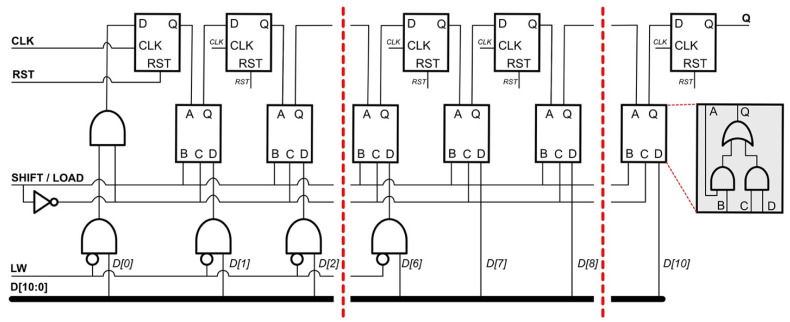
Parallel-to-serial converter of the transmitter.

Figure 3a shows the timing diagram of *V*_REC_, *M_RST*, *BT*, and *FTD*, and Figure 3b–d show the digital controllers PCU, PRCU, and FBCU, respectively, proposed in this work. As discussed in the previous section, the operation of the implanted system relies on a sufficient wireless power transfer efficiency, which is commonly affected by misalignment of the coils between the transmitter and receiver. It is essential to manage the power distribution in the entire block while continuously maintaining its safety level. The PCU shown in Figure 3c plays an important role in tracking the rectified voltage level. As shown in Figure 3a, the master reset signal *M_RST* is triggered when *V*_REC_ is <3.5 V. Consequently, interrupt data are generated at *BT*. The interrupt data composed of the *CLK_S* signal fill the registers in the SP block of the FBCU, as shown in Figure 3a. Because the clock signal of the SP is *CLK_S*, the *Q [0:10]* registers hold data 1. When *V*_REC_ reaches 3.5 V, *M_RST* becomes low, and simultaneously, the *BT* signal becomes low because *CONT* is “0”. *LT* becomes “0” when registers *Q [8]*, *Q [9]*, and *Q [10]* are “0”, “1”, and “1”, respectively. The parallel-to-serial converter circuit is shown in Figure 4. The data *D [10:0]* are loaded in PS when *LT* is “0”. *LW* holds the initial value of “1”, which sets the information data in PS to “0”. When *LT* becomes “1”, the digital ping with a code of [1010000000] is transmitted to the receiver and processed by the SP block in the PRCU.

During this phase, the transmitted data, which are denoted as *FTD*, are fed to the reset pin of the SP block in the FBCU to clear all registers with “0” bits. Clearing the registers is important, because the *FTD* is demodulated in the transmitter and becomes the SP input. In the receiver, the *EN* signal increases when *Q [10:7]* is [1010]. When *EN* = 1, the output data of *Q_TX [6:0]* can be fetched, and wake-up data *SEL* and *CONT* are sent to the PCU. Error data packet #1 is generated when *V*_REC_ is between 3.5 and 5 V. The process continues, and the *BT* data are sent back to the transmitter. In this phase, when the input signal of the D-flip flop is low, LW becomes “0” because the D-flip flop clock in the FBCU is triggered to “1”. Consequently, a data packet containing the header and information is transmitted to the receiver. When *V*_REC_ is >5 V, the bit “0” enters the MUX in the PCU, because *A1* = 1. Under this condition, error data packet #2 is [1110000000], as indicated by the timing diagram.

The *RESTART* pin on the SP block, which is shown in Figure 3a, is used to force the system to execute the start-up operation. Switching it to logic “1” causes the internal registers of SP to be logic “1”. When *RESTART* is switched back to logic “0”, a digital ping is generated and transmitted to the receiver. Table 1 presents the pin functions of the proposed digital controllers.

## 3. Results

**Figure 5:**
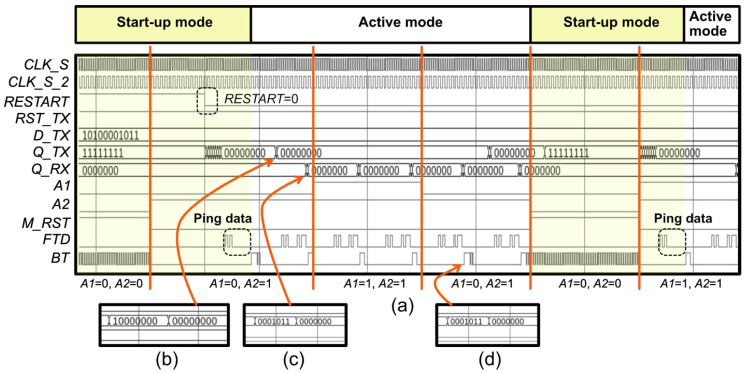
(**a**) Simulation results for the proposed PCU; (**b**) magnified signal of *Q_TX*; (**c**) zoomed signal of *Q_RX*; (**d**) zoomed signal of error data packet #1.

The proposed digital controllers shown in Figure 3 were designed and simulated using the ModelSim tool. *CLK_S* and *CLK_S_2* were set as 2 and 1 MHz, respectively, with a duty cycle of 50%, and *D_TX* was set as [10100001011]. The duration of the Tx data packet (*D_TX*) was 5.5 µs. The simulation started with *A1* = 0 and *A2* = 0 (start-up mode), representing *V*_REC_ < 3.5 V. As shown in Figure 5a, *M_RST* became “1”, and the receiver sent interrupted data to the transmitter. Digital ping data were sent to the receiver when *M_RST* and *RESTART* became “0”. For *A1* = 0 and *A2* = 0, the output of Q_TX was [11111111]. For A1 = 0 and A2 = 1 (active mode), the BT sent error data packet #1 to the transmitter, and Q_TX produced [10000000], as shown in Figure 5b. *Q_TX* output [00000000] when the *FTD* sent data to the receiver, and *Q_RX* produced [0001011] from the *FTD*, as shown in Figure 5c. In the following clock cycle, both *A1* and *A2* were set as “1” (active mode). Thus, *BT* generated error data packet #2 and *Q*_TX output [00000000]. The simulation results confirmed that the operation of the proposed digital controller matched the flow diagram shown in Figure 2.

**Figure 6:**
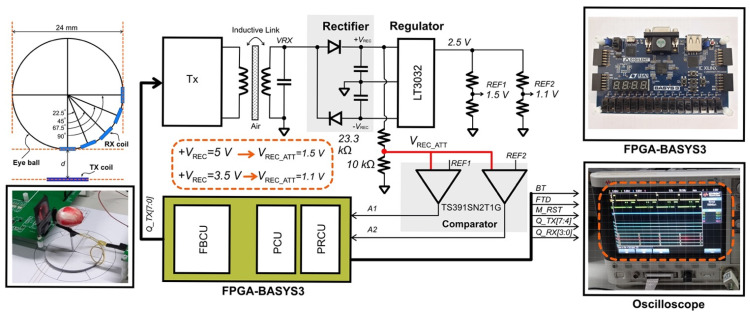
Experimental setup for the proposed digital controllers.

Figure 6 shows the experimental setup for the proposed system, which was composed of a class-E amplifier in the transmitter to wirelessly transmit power through an inductive link. The Tx coil was fixed on a printed circuit board, whereas the Rx coil was attached to an eyeball model with a rotating platform for an angular misalignment test. The received signal, i.e., *V_RX_*, was rectified and then regulated by a low-dropout voltage regulator (LT 3032, Analog Devices, Wilmington, MA, USA) to supply 2.5 V. The reference voltages *REF1* and *REF2*, which were 1.5 and 1.1 V, respectively, were fed into comparators (TS391SN2T1G, Onsemi, East Greenwich, RI, USA). The rail voltage of the comparators was 2.5 V (supplied by LT3032). With the attenuated voltages of 1.5 and 1.1 V, when the *V_REC_* voltages were 5 and 3.5 V, respectively, the input offset voltages in the comparators caused a nonzero input voltage difference, which drove the outputs of *A1* and *A2* to 2.5 V (bit “1”) or 0 V (bit “0”). In the case of zero input voltage difference in the comparator, the output was centered at 1.25 V, corresponding to bit “1”. Furthermore, a field-programmable gate array (FPGA, Basys 3) processed *A1* and *A2.* The programmed FBCU, PCU, and PRCU in the FPGA generated the output signals displayed by the oscilloscope. The PCU generated *BT* and *M_RST* signals, the PRCU generated *Q_RX [3:0]* signals, and the FBCU generated *Q_TX [7:0]* signals.

**Figure 7:**
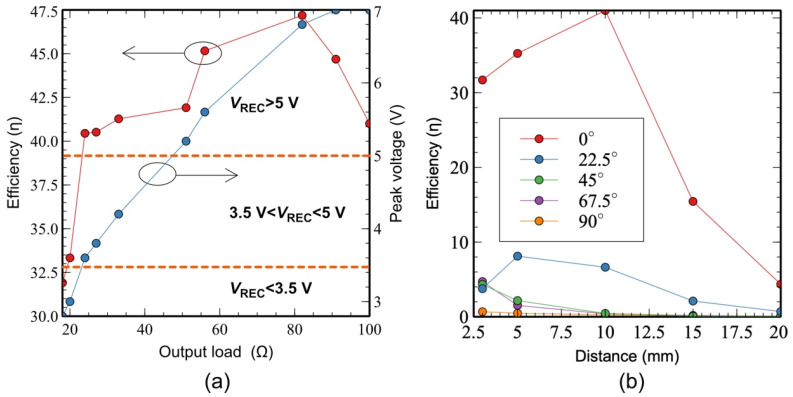
Measurement results for (**a**) the power efficiency and peak voltage with respect to the output load and (**b**) the power efficiency with respect to the distance *d*.

The relationship between the power transfer efficiency and the output load variation was examined, as shown in Figure 7a. The maximum efficiency in this setup was 47% for *R*_L_ = 82 Ω. The peak voltage of the R_X_ signal is shown in the same figure. The minimum peak voltage of the *V*_REC_ required in our system was 3.5 V, which was achieved at *R_L_* = 23 Ω. In this case, a 152 mA current flowed to the load, which was sufficient for our application. Additionally, the effect of the angular misalignment on the power transfer efficiency was evaluated, as shown in Figure 7b. The angle of the R_X_ coil shown in Figure 6 was changed from 0° to 90°, while the distance *d* was changed from 0 to 20 mm. The maximum power efficiency of 40% occurred for *d* = 10 mm, with the T_X_ and R_X_ coils aligned. Significant power loss was observed when the R_X_ coil moved to the 22.5° position, with a reduction of >30% in the power efficiency. To maintain a *V_REC_* of >3.5 V that produces *A1* = 0 and *A2* = 1, the minimum power efficiency of the system should be 33%. This occurs when the Tx coil is perfectly aligned with the Rx coil.

**Figure 8:**
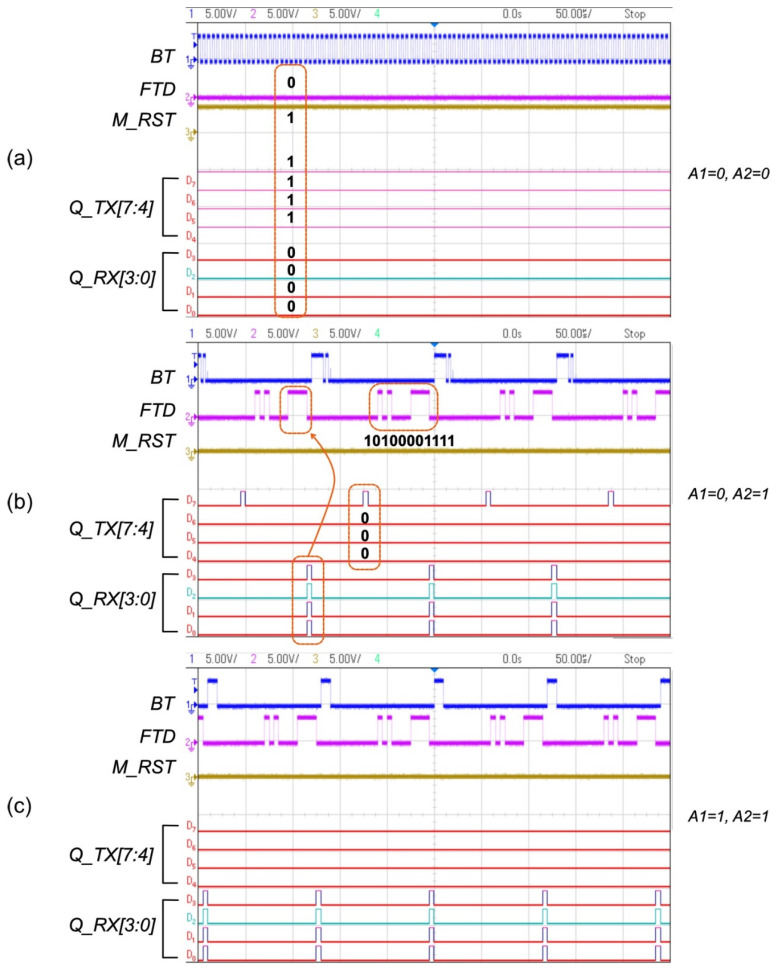
Measurement results for the proposed system: (**a**) *A1* = 0 and *A2* = 0 (*V_REC_* < 3.5 V); (**b**) *A1* = 0 and *A2* = 1 (3.5 V < *V_REC_* < 5 V); (**c**) *A1* = 1 and *A2* = 1 (*V_REC_* > 5 V).

A Basys 3 FPGA board was programmed to verify the functionalities of the proposed digital controllers (FBCU, PCU, and PRCU) when *A1* and *A2* were input to the PCU. The utilization reports of FPGA were tabulated in tables in Appendix A. The transmitter and receiver controllers were implemented in a single FPGA (Basys 3). The measurement results are shown in Figure 8. For all the conditions, the transmitted data, i.e., *D_TX [10:0]*, were set as [10100001111]. In the worst-case condition, as shown in Figure 8a, *Q_TX [7:4]* and *Q_RX [3:0*] became [1111] and [0], respectively, as *FTD* remained at “0”. In the typical case of *A1* = 0 and *A2* = 1, as shown in Figure 8b, *Q_TX [7:4]* changed to [1000], and the RX data of [1111] were captured for *Q_RX [3:0]*. The same Rx data were obtained for the best-case condition, as shown in Figure 8c, while *Q_TX [7:4]* became [0], indicating that the power level of the implanted system was stable. These experimental results agree with the simulation data shown in Figure 5.

## 4. Conclusions

This paper presented two-way communication digital power controllers for wireless retinal prostheses. For implanted retinal devices, coil misalignment after implantation or arising from eye rolling often results in a low power transfer efficiency, causing system breakdown. Thus, power management for retinal prosthetic systems is necessary to ensure their functionality inside the eyeball. The most effective method is to generate a precaution flag before the system becomes faulty by observing the rectified voltage condition. In this work, we proposed three digital power controllers, i.e., an FBCU, a PCU, and a PRCU, to continuously monitor the voltage-level condition, produce a bitstream in the receiver that is sent to the transmitter, and adjust the power delivery of a class-E amplifier in the transmitter. This two-way communication between the transmitter and receiver ensures that sufficient power is transferred to implanted devices.

**Figure 9:**
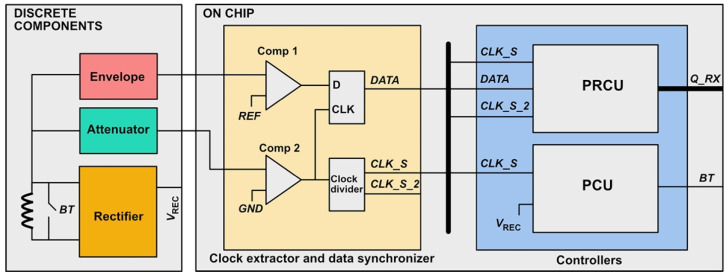
System integration in the receiver.

As shown in Figure 9, a hybrid architecture of the receiver system that includes discrete components and the fabricated chip was proposed, which will be investigated in future research. The PCU and PRCU were fabricated along with a clock extractor and data synchronizer. The attenuator, envelope detection circuit, and voltage rectifier were wirelessly powered through an inductive link. For back telemetry communication, a capacitive load modulation circuit will be added in the near future. Connecting a capacitor in parallel with the coil modulates the impedance observed by the coil, which is then reflected to the primary side. The change in the current in the transmitter coil due to the reflected impedance can be interpreted by the transmitter controller.

## Figures and Tables

**Table 1:** Pin functions of the proposed digital controllers.

Pin	I/O	Description
*CLK*	Input	Main clock source, f_o_ = 13.56 MHz
*BT_DATA*	Input	Back telemetry data
*CLK_S*	Input	*CLK*/n, f_1_ = 2 MHz
*CLK_S_2*	Input	*CLK_S*/2, f_2_ = 1 MHz
*LT*	Input	Load/Shift pulse in Tx SP converter, 0/1 = load/shift
*D [10:0]*	Input	Input data of Tx PS converter *D [10:7]* is header, *D [6:0]* is command data
*Q_TX [7:0]*	Output	Output data of Tx SP converter, [1111111] for VREC < 3.5 V, [1000000] for 3.5 V < VREC < 5 V, [0000000] for *V*_REC_ > 5 V
*FTP*	Output	Transmitted data/reset pulse in Tx SP converter Data speed = 2 Mb/s
*SEL & CONT*	Output	Used to generate *BT* pattern pulses
*BT*	Output	Back telemetry data Data speed = 2 Mb/s
*M_RST*	Output	Master reset
*DATA*	Input	Input data of Rx SP converter Data speed = 2 Mb/s
*Q_RX [6:0]*	Output	Output data to stimulator controller

## Data Availability

The data presented in this study are included in this article.

## Appendix A

Table A1, Table A2, Table A3, Table A4 and Table A5 show FPGA utilization reports.

**Table A1:** Clocking.

bufgctrl_available = 32	bufgctrl_fixed = 0	bufgctrl_used = 1	bufgctrl_util_percentage = 3.13
bufhce_available = 72	bufhce_fixed = 0	bufhce_used = 0	bufhce_util_percentage = 0.00
bufio_available = 20	bufio_fixed = 0	bufio_used = 0	bufio_util_percentage = 0.00
bufmrce_available = 10	bufmrce_fixed = 0	bufmrce_used = 0	bufmrce_util_percentage = 0.00
bufr_available = 20	bufr_fixed = 0	bufr_used = 0	bufr_util_percentage = 0.00
mmcme2_adv_available = 5	mmcme2_adv_fixed = 0	mmcme2_adv_used = 0	mmcme2_adv_util_percentage = 0.00
plle2_adv_available = 5	plle2_adv_fixed = 0	plle2_adv_used = 0	plle2_adv_util_percentage = 0.00

**Table A2:** IO standard.

blvds_25 = 0	diff_hstl_i = 0	diff_hstl_i_18 = 0	diff_hstl_ii = 0
diff_hstl_ii_18 = 0	diff_hsul_12 = 0	diff_mobile_ddr = 0	diff_sstl135 = 0
diff_sstl135_r = 0	diff_sstl15 = 0	diff_sstl15_r = 0	diff_sstl18_i = 0
diff_sstl18_ii = 0	hstl_i = 0	hstl_i_18 = 0	hstl_ii = 0
hstl_ii_18 = 0	hsul_12 = 0	lvcmos12 = 0	lvcmos15 = 0
lvcmos18 = 0	lvcmos25 = 0	lvcmos33 = 1	lvds_25 = 0
lvttl = 0	mini_lvds_25 = 0	mobile_ddr = 0	pci33_3 = 0
ppds_25 = 0	rsds_25 = 0	sstl135 = 0	sstl135_r = 0
sstl15 = 0	sstl15_r = 0	sstl18_i = 0	sstl18_ii = 0

**Table A3:** Memory.

block_ram_tile_available = 50	block_ram_tile_fixed = 0	block_ram_tile_used = 0	block_ram_tile_util_percentage = 0.00
ramb18_available = 100	ramb18_fixed = 0	ramb18_used = 0	ramb18_util_percentage = 0.00
ramb36_fifo_available = 50	ramb36_fifo_fixed = 0	ramb36_fifo_used = 0	ramb36_fifo_util_percentage = 0.00

**Table A4:** Primitives.

bufg_functional_category = Clock	bufg_used = 1	fdre_functional_category = Flop & Latch	fdre_used = 38
fdse_functional_category = Flop & Latch	fdse_used = 1	ibuf_functional_category = IO	ibuf_used = 16
lut1_functional_category = LUT	lut1_used = 3	lut2_functional_category = LUT	lut2_used = 1
lut3_functional_category = LUT	lut3_used = 9	lut4_functional_category = LUT	lut4_used = 2
lut5_functional_category = LUT	lut5_used = 14	lut6_functional_category = LUT	lut6_used = 7

**Table A5:** Slice logic.

f7_muxes_available = 16300	f7_muxes_fixed = 0	f7_muxes_used = 0	f7_muxes_util_percentage = 0.00
f8_muxes_available = 8150	f8_muxes_fixed = 0	f8_muxes_used = 0	f8_muxes_util_percentage = 0.00
lut_as_logic_available = 20800	lut_as_logic_fixed = 0	lut_as_logic_used = 31	lut_as_logic_util_percentage = 0.15
lut_as_memory_available = 9600	lut_as_memory_fixed = 0	lut_as_memory_used = 0	lut_as_memory_util_percentage = 0.00
register_as_flip_flop_available = 41600	register_as_flip_flop_fixed = 0	register_as_flip_flop_used = 39	register_as_flip_flop_util_percentage = 0.09
register_as_latch_available = 41600	register_as_latch_fixed = 0	register_as_latch_used = 0	register_as_latch_util_percentage = 0.00
slice_luts_available = 20800	slice_luts_fixed = 0	slice_luts_used = 31	slice_luts_util_percentage = 0.15
slice_registers_available = 41600	slice_registers_fixed = 0	slice_registers_used = 39	slice_registers_util_percentage = 0.09
lut_as_distributed_ram_fixed = 0	lut_as_distributed_ram_used = 0	lut_as_logic_available = 20800	lut_as_logic_fixed = 0
lut_as_logic_used = 31	lut_as_logic_util_percentage = 0.15	lut_as_memory_available = 9600	lut_as_memory_fixed = 0
lut_as_memory_used = 0	lut_as_memory_util_percentage = 0.00	lut_as_shift_register_fixed = 0	lut_as_shift_register_used = 0
lut_in_front_of_the_register_is_unused_fixed = 0	lut_in_front_of_the_register_is_unused_used = 12	lut_in_front_of_the_register_is_used_fixed = 12	lut_in_front_of_the_register_is_used_used = 8
register_driven_from_outside_the_slice_fixed = 8	register_driven_from_outside_the_slice_used = 20	register_driven_from_within_the_slice_fixed = 20	register_driven_from_within_the_slice_used = 19
slice_available = 8150	slice_fixed = 0	slice_registers_available = 41600	slice_registers_fixed = 0
slice_registers_used = 39	slice_registers_util_percentage = 0.09	slice_used = 24	slice_util_percentage = 0.29
slicel_fixed = 0	slicel_used = 13	slicem_fixed = 0	slicem_used = 11
unique_control_sets_available = 8150	unique_control_sets_fixed = 8150	unique_control_sets_used = 6	unique_control_sets_util_percentage = 0.07
using_o5_and_o6_fixed = 0.07	using_o5_and_o6_used = 5	using_o5_output_only_fixed = 5	using_o5_output_only_used = 0
